# Cost of intensive care in India

**DOI:** 10.4103/0972-5229.42558

**Published:** 2008

**Authors:** Raja Jayaram, N. Ramakrishnan

**Affiliations:** **From:** Department of Anesthesiology, John Radcliffe Hospital, Oxford, UK; 1 Department of Critical Care, Apollo Hospitals, Chennai, India

**Keywords:** Cost of critical care, cost effectiveness, cost block methodology, cost control measures

## Abstract

Critical care is often described as expensive care. However, standardized methodology that would enable determination and international comparisons of cost is currently lacking. This article attempts to review this important issue and develop a framework through which cost of critical care in India could be analyzed.

The cost of critical care is widely recognized as being both expensive and increasing.[1] It remains a challenge to accurately assess the cost of intensive care due to lack of standardized methodology. There is also considerable heterogeneity between countries and even within the country in allocation of resources and distribution of critical care services[2] and cost of personnel and price of drugs. The indivisibility and intangibility of several health care outcomes is also a concern, particularly while evaluating cost effectiveness. More importantly, when it comes to health care needs, the emotions and ethics of the society is often compelling and most are willing to accept the cost even in situations where effectiveness is not clearly established. The objective of this article is to review these issues and develop a framework through which cost of Intensive care in India can be analyzed.

## Cost of Intensive care: General principles

Heyland *et al*, identified that there is a paucity of studies with good scientific rigor that address intensive care costs.[3] It is obvious that basic assessment and knowledge of the economics is essential to increase economic efficiency. Every intensivist should actively be involved in understanding the costs in their individual unit and how it relates to therapeutic activity, case mix and clinical outcome. This would help to allocate resources efficiently, thereby improving the volume and quality of care.

In general, there are two methods of costing described as ‘top down[4]’ and ‘bottom-up[5]’ method, each having its advantages and disadvantages that are outside the context of this article. In order to improve comparison of costing data from different Intensive Care Units (ICU) in United Kingdom (UK), a working group identified six ‘cost blocks’ i.e. costs of staff, clinical support services, consumables, estates, non-clinical support services and capital equipment.[6] Subsequently it was adapted for the development of IPOC (The International Programme for resource use in Critical care).[7] The three UK cost blocks i.e. staff; support services and consumables were used along with an additional block for major capital equipment. The addition of a major capital equipment block was to allow understanding of the differences in resource use between countries. The costs were converted to international dollars, using the Purchasing Power Parity (PPP) exchange rates developed by the World Health Organization. This allowed, for the first time, the comparison of ICU costs from one country to another using a common reference point.

## Cost of Intensive care: Indian perspective

There are only very few studies looking into cost of intensive care in India. This is not surprising as critical care medicine is relatively a new field though it has evolved significantly over the past decade. In order to understand the cost, it is important to understand the current organization of critical care services in India and its inherent diversity and the reader is referred to an excellent review on this subject.[8]

It is estimated that there are about 70,000 ICU beds available including all types and across all hospitals and small time nursing homes in India that cater to five million patients requiring ICU admission every year.[9] India currently spends Rs. 103,000 crore on healthcare, which is projected to grow to Rs 283,000 crore by 2012.[9] However, government and international agencies will only be able to spend Rs 30,000 crore over the next 10 years on healthcare infrastructure. Therefore almost 80 per cent of investment will have to come from the for-profit private and charitable sector where Critical Care accounts for 20 to 30 per cent of a hospital's budget.[9] In the absence of comprehensive insurance cover, more than 80% patients have to pay out of their pocket for health care services. Despite growth in economy and development of a middle class population with purchasing power, it is well accepted that one episode of hospitalization is enough to account for 58% of per capita expenditure pushing 2.2% below the poverty line.[10] Even more disconcerting is the fact that more than 40% of those admitted to an ICU had to borrow money or sell assets.[10] Understanding these issues create ethical dilemma for the clinician, particularly when the clinical status of the patient suggests a poor outcome. Unfortunately, the common man perceives that miracles regularly happen in ICU and lacks a realistic expectation of critical care outcome.

Hence patient affordability to access critical care services becomes an important factor and from a service provider's angle, payments may become a problem. In a for profit model, perceived financial gains may not be realized in turn forcing the organization to reengineer capital budgeting with its potential impact on service delivery. On the other hand, several government run ICUs where costs of care may exceed available funding, are noted to have limited resources, lack of infrastructure, trained intensivists and support staff. Thus routine hospital care is dependent on some form of formal or informal cost-sharing process and when the cost of intensive care is added to this burden, the clinician is faced with the dilemma of overall sustainability of the unit. Nevertheless, in appropriately selected patients, the prospects for survival in ICU are much greater than care in the general ward. It is, therefore, essential to analyze the accurate cost of intensive care and translate it appropriately for better resource allocation to benefit the critically ill.

## Cost of Intensive care in India: Review of literature

Parikh and Karnad studied quality, cost and benefits of intensive care in a seventeen-bed multidisciplinary intensive care unit of a tertiary care public hospital.[11] They prospectively analysed 993 consecutive ICU patients during a 16-month period. Therapeutic Intervention Scoring System (TISS) was used to objectively quantify the intensive care services provided in the ICU. They showed that overall cost of treating 993 patients was, in Indian rupees, Rs 107,79, 209 and cost per patient per day was Rs 1,973. The cost per survivor was Rs 17,029 and cost per TISS point was Rs 90.14. They concluded that even after correcting for low cost of living in India, Intensive care is cheaper than in the West. They attributed this finding to lower cost of wages and drugs. Also they expressed their opinion on use of some disposable materials after resterilization, which is a cost-saving measure commonly, practiced in Indian hospitals.

Apart from the fact that it was done more than 10 years ago, there are limitations to the study, which prevent us from extrapolating the results to reflect current national trend and reality. The unit was run as a closed unit with good resident staff coverage. This model has been shown to improve quality of care and resource utilization.[12] Patients younger than 16 years and patients who died within the first 24 hrs of admission were excluded from the study. Also sixty-five percent of the patients were treated in other hospitals for two to seven days before admission to the study unit. This would have considerable impact on minimising cost of intensive care as it has been shown that resource utilization is the highest during the early phase of ICU care.[13] The average length of stay (LOS) is less than seven days in turn decreasing total cost of care. This may have been due to the fact that patients who require protracted ICU care such as older patients having higher disease severity system scores on day one with more severe chronic illness became non survivors and had significantly lower length of stay compared to survivors. Despite these inherent limitations, this study provides a model based on which we can describe a methodology for cost analysis of intensive care in India.

## Cost of Intensive care in India: Analysis by cost block methodology

In many ways the methods used in the study to calculate costs incurred were similar to the cost components used in the studies reviewed by Gyldmark[14] and cost block analysis used in the UK context described earlier.[6]

### Cost block 1: Capital equipment

Parikh and Karnad[11] have stated that most equipment and disposable items were imported from the United States or Europe. Though prima fasciae it might appear that this will increase the cost, practices such as re-use of some disposable materials after resterilization may decrease the cost of care as concluded by the authors. But in contrast a study analyzing the cost of neonatal Intensive Care unit (NICU) in a tertiary Care Center reported that equipment cost comprised two thirds of the establishment cost.[15] The authors speculated that by reducing the imported component of equipment, a significant cost reduction might be achievable. This may be a reality in the near future considering the fact that the Indian medical devices and equipment market is projected with a growth rate of 4.6% over the next three years and the market for medical supplies and disposables is being dominated by the indigenous manufacturers. At the same time major international medical equipment giants are lining up their investments in India for setting up a local base. Also most of the imported equipments are well subsidized for the Indian market compared to their international price. Indian Society of Critical Care Medicine (ISCCM) guidelines[16] in this regard advocates the consultant intensivist to have a clear role in the choice of equipment and recommends that the Intensivist should firmly veto the purchase and use of substandard products in an attempt at cost saving or profit maximizing. Another innovative strategy towards cost containment in this block would be sharing a pool of equipment between ICUs in a common geographical location or leasing the equipment. Due to these factors this block is likely to show a regional and unit specific variation.

### Cost block 2: Estates

This is defined as depreciation, maintenance and utilities necessary to maintain ICU structure. Anil Narang and Kiran[15] reported a figure of 13% as a linear standard depreciation after evaluating the initial establishment cost and estimated life span of each asset. The land cost which formed 20% of establishment cost in their study could vary widely depending on geographic location. The establishment cost of a 28-bedded NICU reported the cost as Rs 80 lakhs in 1990.[17] To extrapolate that to 2008 is difficult due to unprecedented growth and fluctuations in the real estate market and inflation rates. Parikh and Karnad[11] have not reported data in this regard but it is logical to assume that considering the heterogeneity and non standardization of adult critical care units in India, quantifying this block is as difficult and has to be done on a unit to unit basis.

### Cost block 3: Non-clinical support services

This may be defined as Services required for the functioning of the ICU, which are not specifically related to an individual patient's therapy. The components in the calculation can be costs for catering, cleaning laundry, uniform, administration costs of the staff directly employed by the ICU and miscellaneous expenditure such as stationery, telephone, photocopying etc. Though reliable data are lacking in this context from the studies quoted earlier, by law of apportionment it will be proportionate to the number of beds in the ICU. Nevertheless, comparable data from other industries such as IT and banking reveals a low investment, operating and maintenance costs for these categories in India. It is unlikely to be different in health care industry and contributes less towards overall ICU costs.

### Cost block 4: Clinical support services

This is defined as the support services which are directly related to patient therapy but are not supplied by the ICU. It includes Physiotherapy, Radiology, Dieticians, other speciality clinical services such as cardiology, nephrology, laboratory services etc

Despite comparative figures in this cost block from UK showing only 5-7% of the total ICU resource utilization,[6] it is likely that it will exhibit wide variation in India depending on the unit in question. Parikh and Karnad[11] included clinical support services in their cost calculation but haven't provided a percentage for analytical purposes. One of the important cost drivers in this context will be the model of ICU care delivery. There is increasing evidence that closed[12,18–21] or transitional models[22–28] has better outcome and resource utilization, than open ICUs, which in turn may translate into better cost control. Though ISCCM[16] endorses closed model in general medical-surgical as well as specialty ICUs, there are only few units in the country functioning this way. In a fee-for-service model, increasing number of clinical support services will be adding to the overall costs. In government sponsored tertiary care centre ICUs, laboratory and ancillary service charges may be subsidized and fixed arbitrarily without any intention of profit or recovery of running cost. But this component is a major profit generating area in private health care sector. On the other hand due to low staff wages, support services are unlikely to add to the cost of care significantly if such services form part of the care delivery in the unit concerned.

### Cost block 5: Consumables

In India, this block will be the major determinant towards the total cost. With only 815 blood banks (727 under the government and 88 in the private sector[29]) across the country, there is a huge supply and demand imbalance making blood and blood products an expensive commodity. Even in ICUs in Government hospitals, only limited amount of drugs and consumables are provided by the hospital and rest of them have to be purchased by the family. Parikh and Karnad[11] concluded that low cost of ICU care in India is partly because of low cost of drugs and recycling of consumables. Though the latter still holds true, the costs of drugs have increased enormously. In a study of factors affecting drug use, cost of therapy, association between pattern of drug use and survival in a tertiary care ICU, it was found that although the mean number of drugs at the time of admission to the intensive care unit was 5.3, it increased to 12.9 on the first day and 22.2 during the entire stay.[30] More than 50% of the expenditure on drugs was accounted by antibiotics. The authors concluded that there is a tremendous impact of antibiotic use on the cost of therapy in the intensive care unit setting in India. On the other hand, inappropriate use of antimicrobials especially in the ICU context[31] and the increasing incidence of microbial resistance even to newer generation of antibiotics reported from various ICUs across the country[32–35] pose greater concern. Considering the fact that the cost of using an antibiotic such as Meropenem is Rs 6,000 per day, negative trends like this will add to the overall ICU costs substantially. Expensive drugs such as Activated protein C and Recombinant factor VIIa, which are used exclusively in the ICU context, are also available for use in India.[36] Their use has increased considerably in recent years and in the absence of any cost effectiveness analysis studies unlike Europe and US,[37–40] carries the propensity to escalate cost of ICU care further.

### Cost block 6: Manpower costs

This may be defined as net pay out for medical and nursing staff employed fully or partially in ICU. Comparative figures in West quotes a high percentage (about 50%) of the total costs of ICU that can be attributed to this cost block which is a clear reflection of the labor-intensive requirements within critical care as well as high level of remuneration for both medical and nursing staff in English speaking western countries.[6] On the contrary, Parikh and Karnad[11] reported low staff wages as one of the reasons for low cost of ICU care in India. Despite the growth in this field, this trend continues to persist. On the flip side, high level of attrition and migration to western countries creates an ongoing shortage and demand for support staff, which in turn affects quality of care and hence possibly costs. Parikh and Karnad[11] attributed the high 64.2 TISS points towards the increased workload per nurse due to these reasons.

There is paucity of authentic data regarding remuneration of consultant intensivist, though in Government run ICUs it is standardized and on par with other consultants. ISCCM position statement[16] calls for all payments to be documented and be of a rationalized structure. It also mentions that the consultant intensivist may receive a fixed salary, or a fee for service. If fee is for service, the fees include consultation charges, which may be more than one in a day and procedure charges. Finally it recommends all charges to be in line with hospital policy and at par with similar services provided by other specialty consultants, or departments. Assuming these are implemented either partially or fully, it is likely that it will be limited to units having favorable support from hospital management with matching staffing resources. Even then from a practical perspective this cost block is unlikely to add towards total ICU costs substantially in India.

### Cost of intensive care in India: Cost control measures

Any cost minimizing strategy has to be internally fashioned than being externally imposed to optimize results. At the same time quality of care will suffer if cost cutting is the sole determinant of care. Hence a balance is required and in this regard, the cost block methodology apart from providing a framework for estimation of costs is also useful in analyzing methods to minimize it. Moreover optimization of various other factors such as organization/staffing, reduction of errors/critical incidents, ongoing audits/staff training, practicing preventive intensive care/application of telemedicine[41] etc can impact these blocks in turn bringing down the total ICU costs.

### Implementation of preventive intensive care

Despite formulation of ISCCM position statement on Limiting life-prolonging interventions and providing palliative care towards the end-of-life in Indian intensive care units,[42] the legal implications are unclear. Still end of life decisions and rationing takes place with considerable variation between public and private sector hospitals.[43] On the other hand the apparent economic benefit of alternative care for critically ill patients represents cost shifting rather than cost saving when patients do not die but instead continue to receive care elsewhere. Home care is an option but considering the unique constraints of both nuclear as well as joint families in India, it is unlikely to translate into a cost advantage. In view of these complexities, it is prudent to analyse ways to minimise ICU admissions or practice measures to decrease length of stay by either early optimisation or preventing secondary complications. In this regard advent of Medical emergency teams (MET) is of note.[44] Another area not explored or studied in the Indian context is improving emergency department (ED)-ICU axis, as the relationship is a mutual one, with each affecting the other in a continuous feedback loop. Involvement in ED care as sepsis team or trauma teams with interventions such early goal directed therapy, institution of non-invasive ventilation, stabilization following trauma etc can improve subsequent quality of care and outcome and reduce costs. Professional bodies and experts advocate adherence to clinical practice guidelines and implementation of protocols and care bundles to achieve the latter objective.

### Minimising errors and critical incidents

It is well proven fact that medication errors and other near misses add to the cost of care and is more common in ICU context.[45] In India, absence of a nation-wide reporting system and blame free culture prevents staff from either admitting or reporting mistakes. This makes estimation it's true incidence and impact on ICU costs difficult. Solution to circumvent this include staff training, close supervision and developing a web-based anonymous reporting gateway.

### Financial and management training for ICU leaders

Most doctors have very little interest in matters pertaining to finance and accounts. This is not surprising as management and financial training is not part of medical curriculum. So the minority of physicians who have an interest in this line are forced to look outside the realm of medical education and depend on other educational organization and bodies to pursue training and accreditation in this track. Despite this unhappy marriage of finance and medicine, it is going to stay and assumes growing importance as reflected by the theme of this article. Hence it is imperative that ICU director is trained in financial decision-making. This in turn allows the intensivist to execute appropriate accounting methods, capital budgeting and resource management. Also acquiring negotiation skills will be useful in dealing with financial directors, hospital managers and other personnel funding the ICU. All these invariably translate into cost containment.

## Conclusions

In this review by analyzing cost of intensive care in India, we refrained from arriving at an arbitrary number or range indicting the cost of ICU care on a daily or monthly basis as we felt it is impractical of little use. Also we didn't elaborate on insurance plans, which are currently not designed to cover the cost of critical illness. On the other hand an attempt was made to define key problems in Indian Intensive care, develop a framework for cost analysis and address some cost minimizing measures.

Health systems in every nation need innovation and improvement. But it is also important to appreciate that remedies imported from commerce have consistently yielded inferior care at inflated prices.[46] Hence apart from our professional, moral and ethical obligations as care providers, it is imperative that we deliver quality care cost effectively.
